# Novel Isoprene Sensor for a Flu Virus Breath Monitor

**DOI:** 10.3390/s17010199

**Published:** 2017-01-20

**Authors:** Pelagia-Irene Gouma, Lisheng Wang, Sanford R. Simon, Milutin Stanacevic

**Affiliations:** 1 Department of Materials Science & Engineering & IPPM-UTARI, University of Texas at Arlington, Arlington, TX 76019, USA; 2 Center for Nanomaterials and Sensor Development, State University of New York, Stony Brook, NY 11794, USA; iamxiaogua@gmail.com (L.W.); sanford.simon@stonybrook.edu (S.R.S.); milutin.stanacevic@stonybrook.edu (M.S.)

**Keywords:** sensors, health, flu virus detection

## Abstract

A common feature of the inflammatory response in patients who have actually contracted influenza is the generation of a number of volatile products of the alveolar and airway epithelium. These products include a number of volatile organic compounds (VOCs) and nitric oxide (NO). These may be used as biomarkers to detect the disease. A portable 3-sensor array microsystem-based tool that can potentially detect flu infection biomarkers is described here. Whether used in connection with in-vitro cell culture studies or as a single exhale breathalyzer, this device may be used to provide a rapid and non-invasive screening method for flu and other virus-based epidemics.

## 1. Introduction

Recent outbreaks of infectious diseases have revealed the paucity of rapid noninvasive methods for screening individuals who might have increased risk of exposure to pathogens. The Centers for Disease Control and Prevention (CDC) are now reporting that, in the United States, a more immediate health crisis than the Ebola epidemic in Africa is emerging with the reappearance of the H3N2 strain of the influenza A virus, a recurring strain that has apparently undergone genetic drift in 2014, such that vaccines may offer only limited protection [1]. According to recent reports in the CDC FluView, influenza resulted in over 26 casualties in the pediatric population as of the beginning of 2015, and health care facilities in over 80% of the states have reported significant upsurges in visits to emergent care facilities consistent with epidemic proportions [2]. The course of influenza, which is generally self-limiting but can be especially problematic for the young and the elderly, is marked by a rapid onset of fever and respiratory symptoms, followed by a gradual resolution of these symptoms; early treatment with antiviral medications can limit the severity of the disease. Unlike some bacterial infections, in which toxins produced by the infectious agent cause much of the pathology, many of the symptoms of influenza reflect the host response to the virus, which is marked by the release of pro-inflammatory cytokines that produce the classic constellation of fatigue, lethargy, diffuse pain, and malaise in addition to the early fever. These inflammatory cytokines may be produced in many other infections as well as with certain immune-stimulatory therapies for conditions such as cancer; patients receiving such agents typically report “flu-like symptoms.” Vaccination can induce some degree of systemic inflammation that is part of the immune response, which has led some patients to confuse their symptoms with having contracted the flu from their vaccination. A general pattern of the course of this viral disease, however, is that inflammation develops early and resolves concurrently with diminution of risk of transmitting the infection. 

A common feature of the inflammatory response in patients who have actually contracted influenza, which is mimicked to some extent by individuals who have received a live attenuated virus in a vaccine formulation, is the generation of a number of volatile products of the alveolar and airway epithelium as well as from leukocytes that infiltrate the lungs. These products include a number of volatile organic compounds (VOCs) and nitric oxide (NO). It is especially important that the time course of appearance of these biomarkers of inflammation roughly coincides with the time course of disease symptoms, especially with regard to onset. Regardless of the instrumentation employed to detect these biomarkers, measurements must be made repeatedly on definitively or potentially infected individuals to map the rise and fall of the biomarkers over time. It should be noted that the need to carry out repeated measurements on individuals is justified not only by the already documented distinctive time course of the levels of biomarkers in influenza, but in other viral diseases, even Ebola [3], as well.

A number of reports in the literature [4,5,6] have described the detection of biomarkers of inflammation in the respiratory system using a variety of techniques, but most methods of detection are research methods, such as gas chromatography/mass spectroscopy (GC/MS) or specific ion flow tube/mass spectroscopy (SIFT/MS), that are not amenable to use by personnel without specialized training and cannot be regarded as portable for use in homes or public places where screening of individuals would be of special value. The instrumentation developed by our team, as described below, and in its earlier form in [7], is certainly compact enough to be easily deployed. It has the added advantage of the capacity to transmit data remotely so that more qualified staff could be called upon to assess the significance of digital readings from the sensors. Unlike the signals from other instruments that have been used to profile and quantitate gaseous analytes in breath, which are interpretable only by research personnel, these sensors have both specificity/selectivity and sensitivity, so that the significance of the digital data can be recognized with a high degree of confidence. Nitric oxide, acetone, and ammonia-gas detecting sensors and breathalyzers have been previously developed and described by our research group [7,8,9,10,11,12,13,14,15,16,17,18]. This work focuses on a novel isoprene sensor to be used together with NO and acetone or ammonia in flu virus detection.

## 2. Results and Discussion

### 2.1. Description of the Breathalyzer Instrumentation

#### 2.1.1. Three-Nanosensor Array Microsystem 

A novel concept of a three-nanosensor array system that may potentially serve as a general monitoring tool for flu infection is described here. By interfacing a three-sensor array to a circuit for electrical readout and temperature control, a complete system capable of capturing a single exhaled breath and analyzing it with respect to the relative content of isoprene, ammonia, and NO is described here. Gouma and Stanacevic have developed and demonstrated single-sensor handheld devices for breath analysis that utilize resistive chemosensing technology [8,9,10]. The advantages of these tools have been (i) a low cost of fabrication of the sensor and the device; (ii) the application of nanotechnology that obviates any need of cooling the sensor chamber or impacting the device portability; (iii) the simplicity of sample acquisition (single exhale into a mouthpiece); (iv) the ease of collecting and analyzing the sensing data obtained; and (v) the built-in stability and reliability of the prototypes. 

While single gas sensing elements may be useful in detecting certain diseases (e.g., asthma monitoring based on NO biomarker detection), monitoring viral infections is much more useful if more than one exhaled breath marker is followed simultaneously over time. By using a crystallo-chemical approach, selective interactions between different gases and distinct crystallographic arrangements of metal oxides were utilized in building the sensing systems [11,12,13]. Furthermore, our earlier research showed that it is possible to control the microstructure of nanocrystalline metal oxide films and the operating temperature of the sensor so as to stabilize oxide polymorph phases that are sensitive to only a specific class, or even a single chemical species of gaseous analytes [14]. 

For example, WO_3_ exists in a series of stable solid phases at different temperatures from α phase to ε phase and an unstable hexagonal phase (*h*-WO_3_) [15]. Open structured *h*-WO_3_ nanoparticles were produced by an acid precipitation method [16]. It was found that *h*-WO_3_ is very sensitive to NO_x_ compared to other gases at 150 °C due to the open tunnel structure of *h*-WO_3_. Such selectivity is lost at 350 °C. Instead, the material is very sensitive and selective to isoprene gas at 350 °C.

#### 2.1.2. Novel Isoprene Detector

The isoprene detector is a novel concept. Isoprene: 2-methyl-1,3-butadiene (C_5_H_8_) is a reactive aliphatic hydrocarbon [7] and a VOC marker of the flu. Although more than 1000 kinds of VOCs have been found in human breath, only a few exist in all human bodies. Among them, isoprene is the most common one, which is always present as a precursor of many important organic compounds during the metabolic process.

The acid precipitation method was used to synthesize the *h*-WO_3_ material. This method is somewhat similar to the one used by Gerand et al. in 1979 [17]. A detailed description is as follows: 1.17 g of Na_2_WO_4_·2H_2_O of analytical grade is dissolved in 17 mL of water and the solution is cooled to 10 °C. To this, 8.4 mL of normal hydrochloric acid solution (analytical grade, 18% in excess of equimolar reaction) cooled to the same temperature is added in one dose. The mixture is put back into the refrigerator and allowed to stay for about 20 h. The following reaction occurs:

(1) $${\mathrm{Na}}_{2}{\mathrm{WO}}_{4}\cdot {2H}_{2}O+H^{+}=H_{2}{\mathrm{WO}}_{4}\cdot {2H}_{2}O+{\mathrm{Na}}^{+}$$

After this time, the whole mixture turned to a whitish gel. Then, 110 mL of water was added to the vessel, and the gel and water were lightly stirred manually. By centrifuging the supernatant liquid was removed. Then, 130 mL of water was added to the precipitate, and the steps of light manual stirring, centrifuging, and removal of supernatant liquid were repeated several times to obtain H_2_WO_4_·H_2_O, the precursor of the final *h*-WO_3_ powders. H_2_WO_4_·H_2_O suspensions were passed to hydrothermal dehydration and carried out in Parr acid digestion bombs at 125 °C ± 5 °C. Dehydration under air: furnace temperature: 300–330 °C; annealing time: 90 min.

**Figure 1:**
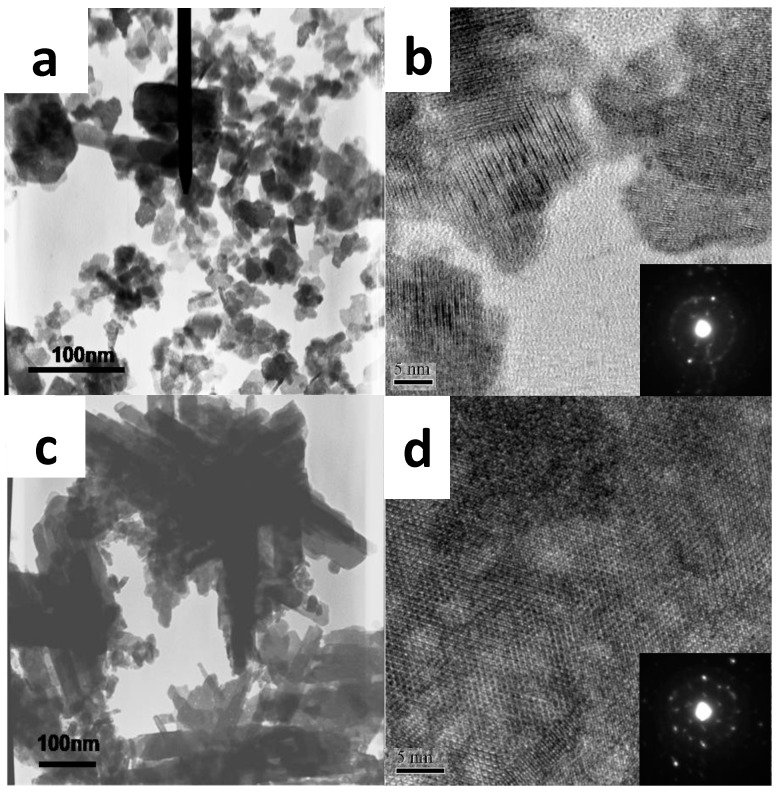
Morphology and structure of *h*-WO_3_ powders: (**a**) TEM image; (**b**) HRTEM image (inset: SAED) of nanoparticles; (**c**) TEM image; (**d**) HRTEM image (inset: SAED) of nanorods.

The color of the synthesized *h*-WO_3_ is grey. There are typically two shapes of grains: equiaxed particles and rod-shaped particles (see Figure 1). These two shapes are mixed together, and the rods are the majority. There is a certain dispersion of the diameter distribution, from 20 to 50 nm, with an average size of 35 nm. The HRTEM image and the SAED pattern (Figure 1c,d) confirm that these particles are polycrystalline and can be indexed in the *h*-WO_3_ structure. The diameters of the rods are 30–100 nm with an average value of 50 nm, and their lengths are up to 100–300 nm with an average value of 200 nm. The HRTEM image clearly records the lattice of the *h*-WO_3_ (001) planes with an interplanar spacing of about 0.39 nm, indicating the WO_3_ rods are single crystalline in most regions and grow along the [001] direction, which is in accordance with the SAED pattern.

**Figure 2:**
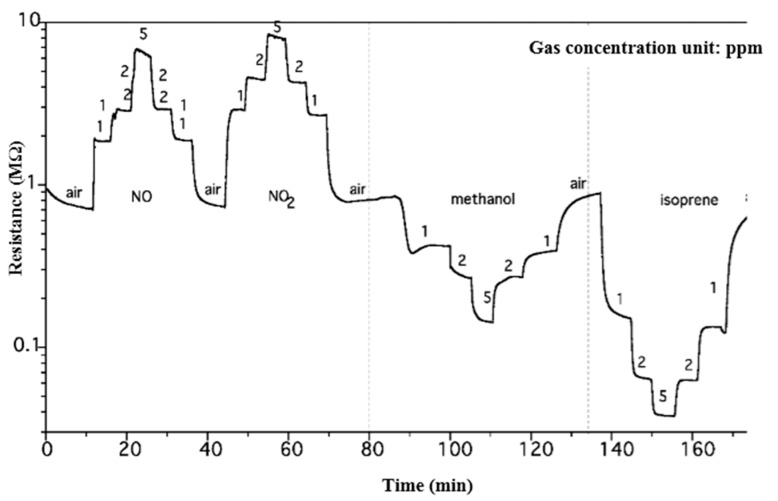
Resistance change of *h*-WO_3_ with exposure to NO, NO_2_, methanol, and isoprene at 350 °C.

A heat treatment was usually necessary for the sensor to become stable, e.g., to remove the residual tungsten hydrate by-product. For *h*-WO_3_, the heat treatment was done at 350 °C for 8 h. *h*-WO_3_ showed a sensitive and selective detection of isoprene at 350 °C (see Figure 2 and Table 1 below). The sensitivity of *h*-WO_3_ sensor to isoprene is 7.34, which is higher than any other gas.

Selective detection on isoprene at 350 °C is clearly proven above. Previous reports on commercially available devices for isoprene detection are always based on spectrometry/spectroscopy methods. Ohira et al. used ozone-induced chemiluminescence as another approach to the measurement of breath isoprene [18]. However, it requires breath sample collection and pre-concentration, making the detection not portable at all. There is a sole study involving isoprene detection using chemo-resistive sensors and that was reported by our group earlier [19]. However, these sensors were based on FSP-made TiO_2_ nanoparticles neither sensitive (*S* = 2, 1 ppm) nor selective (lower sensitivity than acetone) to isoprene. Comparing *h*-WO_3_ to the previously reported *γ*-WO_3_, although *γ*-WO_3_ is also sensitive to isoprene (*S* = 4.8), it has a cross sensitivity to acetone (*S* = 4). Therefore, to the best of our knowledge, the *h*-WO_3_ presented here might be the first isoprene-selective chemical sensor that has the potential to be used in a non-invasive portable device.

#### 2.1.3. The Breathalyzer Device

**Figure 3:**
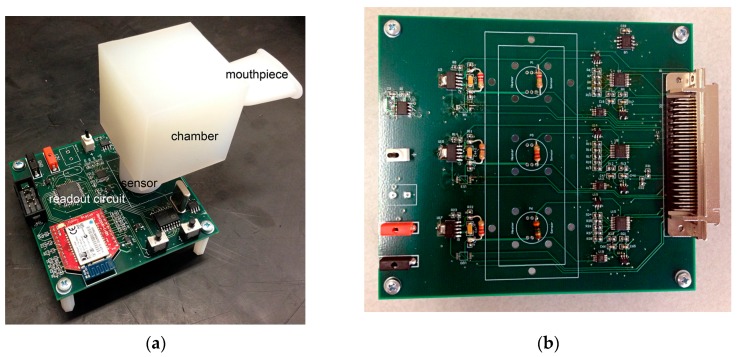
(**a**) A single sensor readout circuit with Bluetooth module; (**b**) A three-sensor system with integrated readout and heater control circuit as a step toward wireless handheld multi-sensor breathalyzer.

We have previously designed a single-sensor system with a Bluetooth interface on a printed circuit board (PCB) with dimensions of 3 × 3 in. (Figure 3a). A three-sensor system with the same readout and heater control circuit with the wired connection to PC has been designed here (Figure 3b). To achieve higher sensitivity in quantifying the gas concentrations and to reduce power consumption, a modification of our previously described single channel readout integrated circuit with CMOS technology was implemented [20]. Although the sensors have high specificity, the digital sensor data from the readout circuit may be further processed to improve selectivity and compensate for cross-sensitivity, using an algorithm to compensate for any drift in the resistance of the sensors over time.

### 2.2. Proposed Use of the Breathalyzer in In-Vitro Studies

#### Limitations of In-Vitro Studies with a Live Attenuated Influenza Virus (FluMist^®^)

The literature on in vitro studies with human cells exposed to influenza virus strains is not extensive. Experiments in which culture medium and/or headspace gas have been assayed subsequent to exposure of mouse neutrophils [21] or an immortalized human B cell line [22] to active influenza virus have been reported in which generation of NO or VOCs was detected in a virus-strain and dose-dependent fashion, but these studies did not effectively replicate the contribution of the epithelium to the environment of the human respiratory system. Studies employing human volunteers exposed to a live attenuated influenza virus vaccine (FluMist^®^) have been carried out, using GC/MS [5] and SIFT/MS measurements, and they show both increases as well as decreases in the levels of a number of VOCs over the course of a two-week period, which the authors have attributed to a combination of the activation of oxidative stress pathways and the inhibition of the formation of lipid peroxidation products (NO levels along with VOC levels were measured in only one study on human subjects [4]). Interpretation of the experimental data required the use of sophisticated instrumentation as well as mathematical models for analysis, making the approach not especially user-friendly, and the use of human subjects imposes significant restrictions on further investigative experiments. The 3-sensor microsystem-based tool described here may provide a viable and inexpensive alternative to carrying out these cell culture studies.

## 3. Conclusions

An unanticipated serious epidemic of influenza can highlight an urgent need facing the population. A strategy to address such a rapidly emerging public health crisis as well as recurrent events that are likely to appear in the future is needed. One viable approach makes use of a system that can monitor the ongoing progression of individual cases of influenza or other diseases with a major respiratory component so that persons with symptoms of lung inflammation can be properly managed with therapeutic intervention or supportive care. The system described here relies on a device that can measure biomarkers of the inflammatory response in breath, noninvasively. The sensor system utilizes a 3-probe array to detect nitric oxide (NO) and volatile organic compounds (VOCs) and may be used by personnel with minimal training to collect and transmit data from individuals suspected of early stages of illness to central facilities, where more medically skilled workers can call for early intervention. We envision that, in its final iteration, our system could be taken home by a patient who could then simply exhale into the sensor system to generate the relevant data that would then be transmitted to a central service for collection and interpretation.

## Figures and Tables

**Table 1:** Response of *h*-WO_3_ to various gases of 1 ppm concentration at 350 °C.

	Sensitivity	Response Time (s)	Recovery Time (s)
NO	2.66	24	98
NO_2_	2.98	82	77
methanol	2	112	380
isoprene	7.34	65	145
